# Human Leukocyte Antigen Typing Using a Knowledge Base Coupled with a High-Throughput Oligonucleotide Probe Array Analysis

**DOI:** 10.3389/fimmu.2014.00597

**Published:** 2014-11-27

**Authors:** Guang Lan Zhang, Derin B. Keskin, Hsin-Nan Lin, Hong Huang Lin, David S. DeLuca, Scott Leppanen, Edgar L. Milford, Ellis L. Reinherz, Vladimir Brusic

**Affiliations:** ^1^Cancer Vaccine Center, Dana-Farber Cancer Institute, Harvard Medical School, Boston, MA, USA; ^2^Department of Computer Science, Metropolitan College, Boston University, Boston, MA, USA; ^3^Department of Medicine, Harvard Medical School, Boston, MA, USA; ^4^Laboratory of Immunobiology, Department of Medical Oncology, Dana-Farber Cancer Institute, Boston, MA, USA; ^5^Institute of Information Science, Academia Sinica, Taipei, Taiwan; ^6^Department of Medicine, Boston University School of Medicine, Boston, MA, USA; ^7^Agilent Technologies, Inc., Santa Clara, CA, USA; ^8^Histocompatibility and Tissue Typing Laboratory, Brigham and Women’s Hospital, Boston, MA, USA

**Keywords:** HLA typing, HLA disease association, population typing

## Abstract

Human leukocyte antigens (HLA) are important biomarkers because multiple diseases, drug toxicity, and vaccine responses reveal strong HLA associations. Current clinical HLA typing is an elimination process requiring serial testing. We present an alternative *in situ* synthesized DNA-based microarray method that contains hundreds of thousands of probes representing a complete overlapping set covering 1,610 clinically relevant HLA class I alleles accompanied by computational tools for assigning HLA type to 4-digit resolution. Our proof-of-concept experiment included 21 blood samples, 18 cell lines, and multiple controls. The method is accurate, robust, and amenable to automation. Typing errors were restricted to homozygous samples or those with very closely related alleles from the same locus, but readily resolved by targeted DNA sequencing validation of flagged samples. High-throughput HLA typing technologies that are effective, yet inexpensive, can be used to analyze the world’s populations, benefiting both global public health and personalized health care.

## Introduction

The human leukocyte antigen (HLA) genomic region contains 12 protein coding genes (HLA-A, B, C, DRA1, DRB1, DRB3, DRB4, DRB5, DQA1, DQB1, DPA1, and DPB1) and over 7,000 allelic variants that regulate immune responses and other important molecular and cellular processes (1). HLA is under a strong selection pressure in human beings, and is subject to rapid genetic divergence to afford protection of interbreeding populations from the emerging or pandemic diseases (2). Associations between HLA and many diseases have been established (1, 3–7). HLA is one of the most critical biomarkers in humans of broad relevance for transplantation (3), transfusion medicine (4), cancer (6), and identification of drug toxicity (7). The HLA region encoding classical transplantation genes is the most diverse within the human genome. The resulting complexity limits our current ability to perform related large-scale population studies. Accurate population-based HLA typing will enable the development of new methods for risk assessment, early diagnosis, prognosis, and optimization of therapies for many diseases.

Current HLA typing uses PCR amplification with sequence-specific oligonucleotide probes (SSOP) in combination with sequence-specific primer (SSP) testing and DNA or RNA sequencing. The SSOP method requires a large number of probes and a series of separate hybridization reactions. These probes may be arranged into arrays to be used in Luminex-based HLA typing (8). DNA microarrays were reported as a variant of the SSOP method (9–11), but have not been used in clinical typing. SSP methods rely on a “yes/no” signal for amplification based upon pairs of PCR primers that detect one or two informative single nucleotide polymorphisms (SNPs) for each reaction. A large number of reactions and primer pairs are necessary to include or exclude known HLA alleles. Sequencing methods deploy PCR amplification of target loci followed by DNA sequencing. Recently, next generation sequencing methods have been used for ultra-high resolution HLA typing using both DNA (12) and RNA (13) sequencing. The standard RNA-Seq approach used for HLA typing showed limited sensitivity (94%) (14). DNA-based HLA typing approaches are currently the methods of choice, but they are labor intensive and are usually limited to typing exons 2 and 3. RNA-based approaches offer a simpler alternative to genomic DNA sequencing as they focus on transcripts that reveal gene expression levels. RNA-based methods can be automated and are more cost effective than DNA sequencing (15).

Ultra-sensitive and highly parallel methods allow for concurrent typing of multiple samples and are amenable to typing of large number of individuals. The technologies are currently rapidly evolving requiring constant updating of reagents, sample preparation, and methods refinement. Ideal HLA typing method must yield a rapid turnaround, be highly accurate, and cost affordable. To ensure standardization, the process should be automatable using robotics for sample preparation and processing (13). Here, we describe a high-throughput method for HLA-A, -B, and -C typing that utilizes hundreds of thousands of SSOPs that completely cover the vast majority of HLA alleles observed in human population.

## Materials and Methods

**Figure 1:**
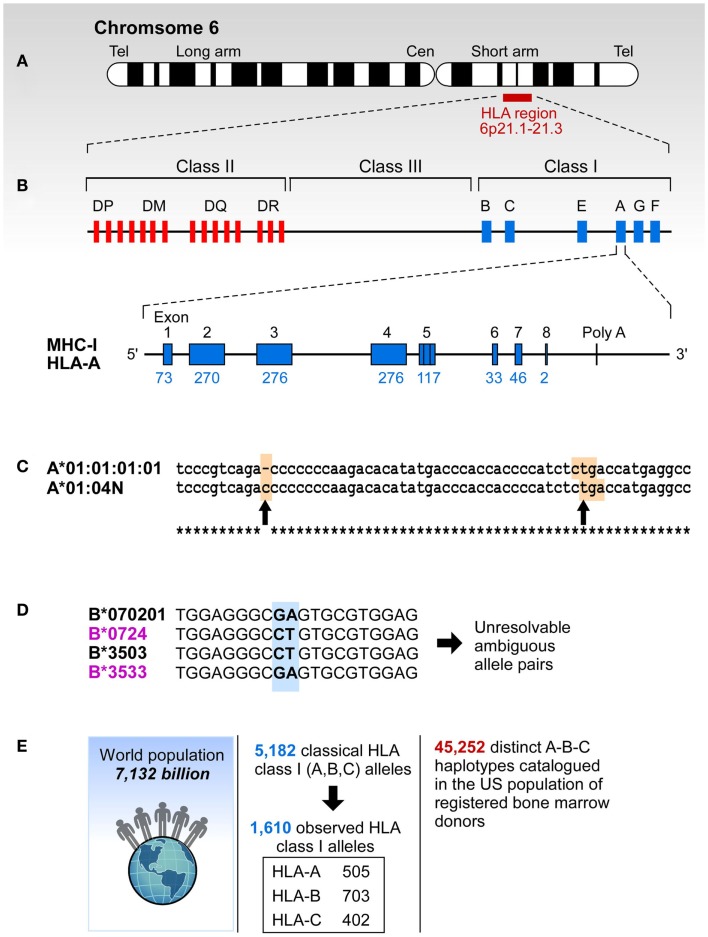
**The complexity of study of HLA**. **(A)** HLA resides at the short arm of human chromosome 6 spreading over approximately 4 million base pairs. **(B)** Class I region has three classical loci (A, B, and C loci) and each of them has eight exons. Each individual has two copies of each locus. **(C)** Sometimes HLA genes are mutated to produce null alleles resulting in non-functional protein products. The example A*01:04N shows an insertion of C in codon 186 (left arrow) that caused a frameshift resulting in a premature in frame stop codon (right arrow). **(D)** HLA alleles are highly similar, and typically have >85% identity across A, B, and C loci. The B7/B35 example shows ambiguity – when two probes are positive, the result is ambiguous. In this case both B*07:02/B*35:03 and B*07:24/B*35:33 are possible. However, B*07:24 and B*35:33 alleles are rare, they have been observed in frequencies of >0.0001% making it highly unlikely that this combination is present in any one individual. On the other hand B*07:02 and B*35:03 have been observed in all of the 22 populations screened in the NMDP and their frequencies range from 0.8-13.1% (B*07:02) and 0.04-7.2% (B*35:03). **(E)** More than 5,000 classic HLA Class I alleles have been identified in the World population. The exclusion of null alleles, other problematic alleles, and low-frequency alleles resulted in 1,687 clinically relevant HLA class I alleles. These alleles combine into >45,000 A-B-C haplotypes.

Most of HLA diversity is encoded by the class I region – more than 5,500 protein variants that code HLA-A, -B, and -C molecules have been reported (16). About 230 Class I HLA alleles are present in the human population at allele frequencies of >0.01% and 111 cover >99% of global HLA alleles present in human population (16, 17) (Table 1). The combinatorial complexity of HLA makes its study difficult (Figure 1). The set of HLA alleles that were arrayed on the HLA chip includes 505 genetic HLA-A, 703-B, and 402-C variants encoding 159, 281, and 123 respective protein variants.

**Table 1:** **The count of HLA genes, proteins, null alleles, and their prevalence in the World’s population**.

Numbers of named HLA alleles (as of February 2014)				
Observed	Class I loci	Genes	Proteins	Nulls
All	A	2,432	1,740	117
	B	3,086	2,329	101
	C	2,035	1,445	57
In population	A		733	24
	B		921	9
	C		429	7
90% Coverage of global HLA alleles	A		15	
	B		33	
	C		13	
95% Coverage of global HLA alleles	A		22	
	B		47	
	C		17	
99% Coverage of global HLA alleles	A		28	
	B		63	
	C		20	

*About 99% of HLA present in human population are covered by only 111 HLA alleles (1,17). Only 230 protein variants of HLA Class I are present in global population at frequencies >0.01% (data not shown)*.

### Alleles, samples and probes

The total number of named HLA alleles includes >7,500 genetic sequences and > 5,500 proteins (Table 1). Alleles included in the microarray design are shown in Table S1 in Supplementary material. All control samples are shown in Table S2 in Supplementary material. The numbers of probes that correspond to HLA-A, -B, and -C alleles are shown in Table 2. The set of HLA alleles that were arrayed on the HLA chip includes 505 genetic HLA-A, 703-B, and 402-C variants encoding 159, 281, and 123 respective protein variants. The sequences of these HLA variants were taken from the HLA-IMGT database (18).

**Table 2:** **HLA class I coverage of the HLA microarray with probe numbers**.

Locus	HLA-A	HLA-B	HLA-C	HLA-E, -F, -G	Total
Number of arrayed sequences	505	703	402	77	1,687
Number of probes	36,003	36,651	28,320	6,771	91,661
Number of unique probes	11,980	12,216	9,432	5,222	33,469

### Sample preparation and processing

**Figure 2:**
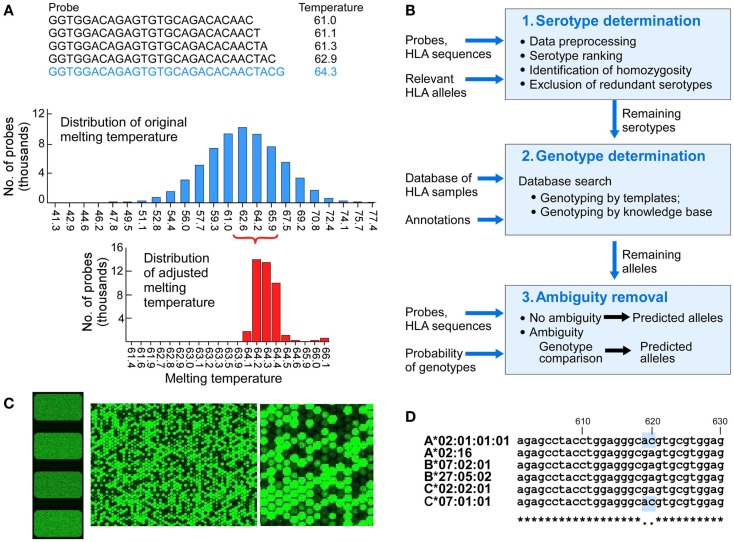
**The design of components of the HLA typing system**. **(A)** Probe design and matching melting temperatures were calculated for each of the 75,476 probes (**A**, upper panel), melting temperatures of the initial probes that were 25 nucleotides long (**A**, middle panel), melting temperature for adjusted length probes targeting 64.2°C (**A**, lower panel). **(B)** Physical design of the microarrays – each slide has four identical arrays, each having a full set of probes covering full length of 1,687 Class I HLA sequences (left image). The two images on the right show increasing magnification of signals from fluorescent signal hybridization. The Agilent Feature Extraction program was used to convert the fluorescence intensity of scanned arrays into text Cy 3 signal files. The Cy3 signals from hybridized high intensity fluorescent samples were measured from the DNA probes organized as spots or “features” on the array. At higher magnification the presence or absence of fluorescent cRNA hybridization is evident (**B**, middle and right images showing signals on a portion of one array). **(C)** Analytical workflow used templates of known samples or a theoretically derived knowledgebase. The three main steps include serotypes determination, genotypes determination, and ambiguity removal. The input includes probes, probe signals, and HLA sequences of target alleles. The output includes genotypes assigned by template matching (see Figure 3 for details) or genotype matching with ambiguity removal (described in the main text). **(D)** Sample 4 (Table 3) was HLA-A*02:01/A*02:01, B*07:02/B*27:05, C*02:02/C*07:01 (A*02:01 homozygous). Probe signal analysis alone could not distinguish whether DFCI-06 is heterozygous for HLA-A*02:01/02:16 or homozygous for either of these alleles because this probe for A*02:01 is identical to (i.e., masked by) C*07:01. Likewise, A*02:16 is masked by its associated HLA-B*07:02/B*27:05/C*02:02 alleles. This issue can be resolved by a single Sanger sequencing run for HLA-A2 locus around positions 610-630.

The preparation and processing steps included RNA extraction, cDNA synthesis, cRNA synthesis with amplification, cRNA purification, preparation of hybridization samples, hybridization, wash, scan, and feature extraction. The RNA from each sample was extracted using the QIAGEN RNeasy Mini Kit. Quality control was performed with the Agilent RNA 6000 Nano Kit. Fluorescent cRNA samples for hybridization were synthesized from 50 ng total RNA using the Agilent single color direct labeling kit in which cRNA was synthesized in the presence of Cy3 conjugated NTPs. Samples were hybridized to custom slides at 64°C for 17 h, based on the Tm of 64.2°C. The slides were scanned using an Agilent Microarray scanner. The signals from high intensity fluorescent cRNA probes were measured from the spots shown in green (Figure 2B). Sample preparation and processing and subsequent computational analysis were performed strictly in a double-blinded fashion.

**Table 3:** **Blood samples used in this study**.

Sample	DFCI-ID	HLA-A	HLA-A	HLA-B	HLA-B	HLA-C	HLA-C	Sample type	Status/actual
1	02	01:01	24:02	08:01	27:05	02:02	07:01	Blood	OK
2	04	03:01	30:01	35:03	Nil	04:01	Nil	Blood	OK
3	05	01:01	24:02	27:07	52:01	12:02	15:02	Blood	OK
4	06	02:01	02:16	07:02	27:05	02:02	07:02	Blood	A*02:01/02:01
5	08	02:06	30:01	13:02	35:01	03:03	06:02	Blood	OK
6	09	01:01	Nil	07:02	08:01	07:01	07:02	Blood	OK
7	10	02:01	31:01	07:02	51:01	15:02	15:05	Blood	OK
8	11	24:02	66:01	27:05	44:02	01:02	05:01	Blood	OK
9	12	02:01	30:02	15:01	50:01	04:01	Nil	Blood	OK
10	13	11:02	33:03	44:03	52:01	12:02	14:03	Blood	OK
11	14	02:01	Nil	18:01	40:02	02:02	07:01	Blood	OK
12	15	01:01	11:01	07:02	52:01	07:02	12:02	Blood	OK
13	16	33:03	Nil	44:03	Nil	07:01	14:03	Blood	C*07:06
14	17	02:01	33:03	40:06	58:01	03:02	08:01	Blood	OK
15	18	24:02	31:01	46:01	52:01	01:02	12:02	Blood	OK
16	19	02:01	03:01	07:02	50:01	06:02	07:02	Blood	OK
17	20	11:01	33:03	15:02	58:01	03:02	08:01	Blood	OK
18	67	11:02	33:03	35:01	44:03	03:03	14:03	FB 1	OK
19	66	31:01	33:03	40:01	44:03	07:02	14:03	FB 2	OK
20	68	11:02	33:03	35:01	44:03	03:03	14:03	FB 1 repeat	OK
21	75	31:01	33:03	40:01	44:03	07:02	14:03	FB 2 repeat	OK

*Blood samples are either newly collected blood (Blood) or frozen blood (FB). The FB samples have two replicates taken at different points in time (12 and 24 months earlier). HLA-A, -B, and -C show assigned alleles. The correct HLA typing is in black font, the incorrect typing is shown in red. The actual alleles, if incorrectly assigned, are shown in column Status/Actual, or designated “OK” if correct*.

### Signal processing – normalization, error correction and probe analysis

Following hybridization, arrays were scanned using Agilent high-resolution GeneArray scanner at 2 μm resolution. The initial processing used Agilent Feature Extraction Software (http://www.chem.agilent.com/library/usermanuals/public/g4460-90039_featureextraction_user.pdf). The raw data signals showed significant variability between individual slides. The ranges of the raw individual array signals varied widely (Min to Max) from 2 to 47,174 or from 2 to 770,759 (Table S3 in Supplementary material). Signal preprocessing included three steps: normalization, error estimation, and error correction. Data normalization ensured that signals from all arrays are mutually comparable. The normalization that mapped signals to a scale of 1–20,000 with average signal being 1,000 was performed – the minimum was set to 1, maximum to 20,000, and the array-wide average to 1,000. Signals from each array were subjects to several transformations. First, we calculated the minimum raw probe signal of the entire array, Rmin. Then we selected a scaling factor *F*, to ensure that the average of the normalized signals is 1,000. A probe raw signal is designated as *S* and its normalized signal as Sn. The normalization was a linear transformation of all signals to the scale 1–20,000 using formula:

$$\mathrm{Sn}=\frac{\min\left(S-\mathrm{Rmin}+F,F\times 20000\right)}{F}$$

Error correction was performed using detection of outliers and correction of noisy signals. The analysis of overlapping probes within a given window showed that the positive signals normally show smooth change between consecutive probes, and negative signals have low values. Positive signals were defined by an allele-specific threshold. Initially, the threshold was set as 10% of maximal signal for a given probe. With the accumulation of data, the threshold was determined empirically from the measurement of specific probes and integrated in the knowledge base (Figure S1 in Supplementary material).

Duplication of probes on the array enabled us to assess the occurrence of random errors and patterns of random errors. Uninformative probes were identified by comparing the signals of each probe across the 60 arrays.

### Computational analysis

For each array, we used a computational approach to analyze all probe signals to determine the most likely HLA Class I genotypes. The computational approach involves three steps: serotype determination, genotype determination, and ambiguity removal. In serotype determination, all serotypes were ranked based on the average number of negative signals for each individual allele. Serotypes that are present in a given sample tend to have smaller average number and an absent serotype by contrast tends to have larger average number of negative probes. Based on this analysis, we could exclude a large number of serotypes that were actually absent. However, not all absent serotypes have a larger number of negative signals because of probe masking (a probe may be present because it is shared with other allele present in the sample, Figure 2D). In genotype determination, we performed both serotype comparisons and allele comparisons to select alleles that are most likely to be present. In cases where there were multiple possible alleles after serotype/allele comparisons, we performed genotype comparison to remove the ambiguities. The workflow for the computational analysis is shown in Figure 2C.

#### Serotype determination

The goal of serotype determination was to maximally reduce the search space by eliminating irrelevant serotypes and ensure that this step does not produce any false negatives. The procedure involved four steps and is performed separately for each of the loci (HLA-A, -B, and -C). These steps were data preprocessing, serotype ranking, homozygotes determination, and redundant serotype reduction.

##### Preprocessing

To efficiently assign HLA Class I genotype, there were 1,687 clinically relevant alleles in total (505 HLA-A, 703-B, and 402-C alleles). These alleles were covered by 33,469 unique probes. Each probe covered a stretch of 20–60 nucleotides from and its signal was assigned to the starting position. We made a multiple sequence alignment for all the 1,687 allele sequences, to help calculate the signal threshold at each position for HLA-A, -B, and -C alleles, respectively.

##### Serotype ranking

The 563 sequences representing HLA-A, -B, and -C alleles were grouped into 21 HLA-A serotypes, 36-B and 14-C serotypes. The Neg(*x*) represent the number of negative probe signals for allele *x*, and Neg(ST) represent the average number of negative probe signals for serotype ST. We ranked all Neg(*x*) that belong to same serotype ST in ascending order and selected k alleles to calculate Neg(ST).

Neg(ST)=∑Neg(x)∕k,

where *k* = Max(5,*N*),*N* is the number of alleles in serotype ST.

When we applied this equation to all present and absent serotypes among the samples, we found that Neg(ST) can effectively differentiate present from absent serotypes. Most of the present serotypes in the samples rank the first or the second in each locus.

There were 472 present serotypes among the samples and 241 of them ranked the first, and 155 ranked the second, while 76 ranked lower (Table S4 in Supplementary material). From the analysis of serotype ranking, we noted that most serotypes ST have a clear boundary of Neg(ST) that distinguishes presence and absence, while some serotypes do not have a clear boundary due to probe masking or because they belong to the same supertype. For example, Figure S2 in Supplementary material shows the distributions of Neg(A*01) and Neg(A*03). A*01 has a clear boundary between A*01 + (present) and A*01 − (absent). However, some arrays with absent A*03 have a lower number of negative signals due to probe masking when A*11 is present in that sample. The average sequence identity between A*03 and A*11 is 98.9% and they share a large number of probes. This is consistent with the taxonomic classification that shows close relatedness of HLA-A*03 and -A*11, while A*01 is well separated from other serotypes (19). Some serotypes, such as A03/A11, A26/A34/A43/A66, A23/A24, and A31/A33/A74 show high sequence similarity. Therefore we analyzed the Neg(ST) distribution of present and absent serotype ST and define a MaxNeg(ST) to indicate the maximal Neg(ST) for a present ST. The MaxNeg(ST) for all serotypes ST is shown in Table S5 in Supplementary material.

##### Homozygocity

Homozygosis checking is an essential step for HLA genotyping. In this study, we determined whether a locus is homozygous or not based on the comparison of the best two serotypes. We designated ST1 as the first ranking serotype of a particular HLA locus L, and ST2 the second ranking. The locus L is considered to be homozygous if Neg(ST2) − Neg(ST1) ≥ 20. In that case, we only keep ST1 and remove all the rest serotypes of the locus L.

##### Redundant serotype elimination

If more than two serotypes remain in the candidate list, we further remove serotypes by using serotype comparison rules. In most cases, redundant serotypes occur because of probe masking. We found that some serotypes normally remain in the list in groups, for example, a relatively well-distinguished A*01 and A*36. If one of them is present, the other will be kept due to their sequence similarities. Therefore, we derived serotype comparison rules to deal with this ambiguity. Given two serotypes, ST1 and ST2, we compare Neg(ST1) and Neg(ST2) and remove ST2 if Neg(ST2) − Neg(ST1) > MaxDifference(ST1, ST2) and ST2 ranks behind the second place. If the rule criterion is not met, it implies that either both ST1 and ST2 are present, or they cannot be differentiated at the serotype level. Serotypes listed in Table S6 in Supplementary material usually cannot be differentiated at the serotype level and are further analyzed. The remaining serotypes ambiguities (more than two serotypes for each locus), if any, are further analyzed in Genotype Determination step.

#### Genotype determination

**Figure 3:**
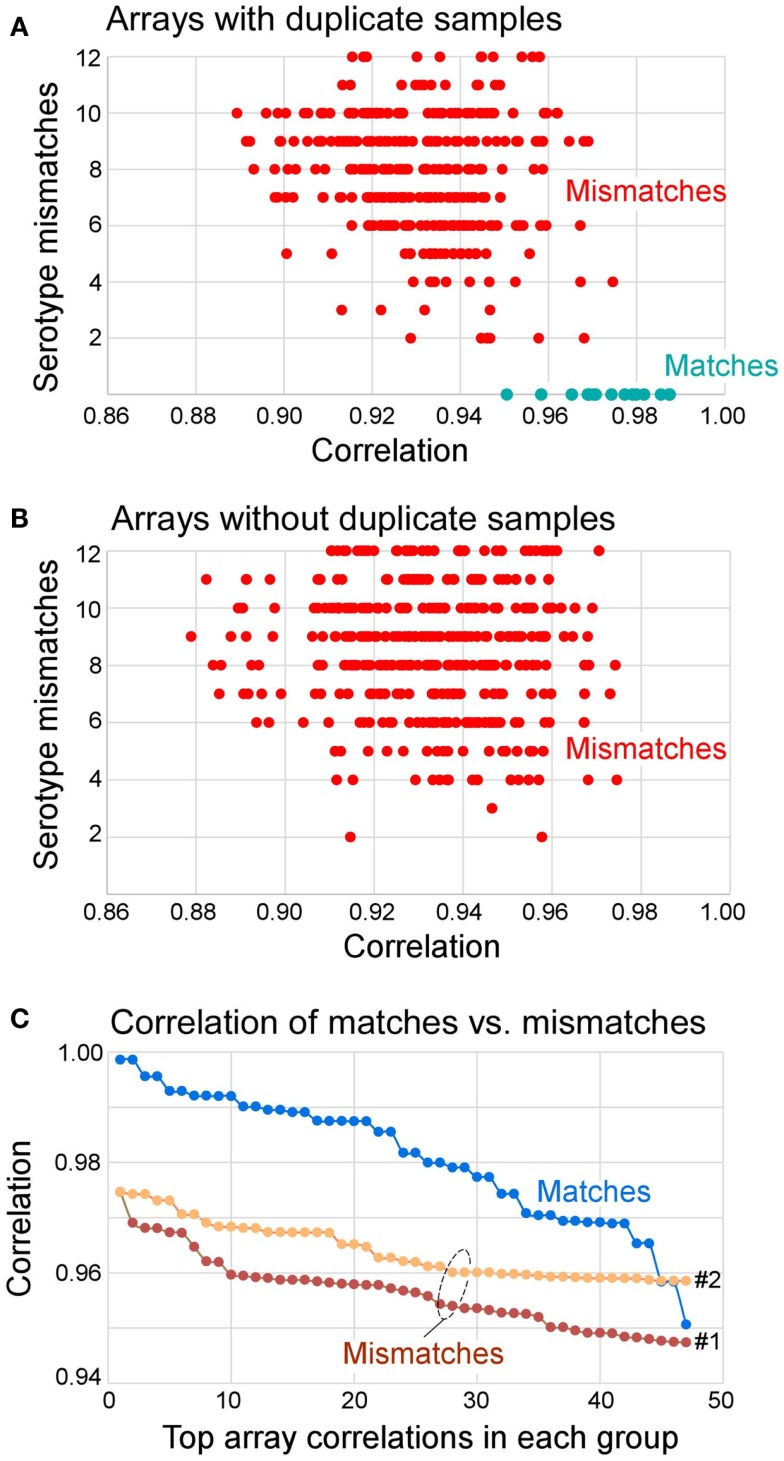
**Distribution of correlation coefficients between matched and mismatched pairs and the number of serotype mismatches**. **(A)** Arrays that had duplicate samples in the knowledgebase (also see Table 5) paired with all other samples. The results show that all matches had zero serotype mismatches. All mismatches had 2–12 serotype mismatches. **(B)** Arrays that did not have duplicate samples in the knowledgebase of templates all had 2–12 serotype mismatches. **(C)** Distribution of top 47 correlation coefficients segregated by matches, mismatches to repeat samples (Mismatches 1, of 360 total), and mismatches of all other samples (Mismatches 2, of 527 total). The Mismatches 1 correspond to data shown in (**A)** and Mismatches 2 correspond to data shown in **(B)**. In summary, 70% of matches can be determined from correlation coefficient alone (*r* > 0.975), and 100% of matches had no serotype mismatch. If *r* > 0.95 and serotype mismatch number is 0, the identity of the query array to the matching template from the knowledgebase can be established.

The most probable alleles are determined from the remaining serotypes. The genotyping was achieved by the comparison with the templates in the knowledgebase (Figure 3), or when unavailable, using the knowledge-based approach where theoretical patterns of negative probes were compared to the corresponding signals. In genotype determination, we combined serotype comparisons and allele comparisons to select alleles that were most likely present. When multiple alleles were possible after serotype and individual allele comparisons, we performed genotype comparison to remove the ambiguities. To apply these methods, we first introduced the comparison vectors for serotypes and alleles, and the comparison algorithm.

##### Serotype and allele comparison

The Serotype comparison vector is generated using the number of wins as we compare positive and negative signal probes. The generation of an allele comparison vector is similar to that of serotype comparison vector though the difference is we compare alleles within the same serotype and determine the most likely allele by the number of wins. Allele comparison algorithm is used to estimate the number of wins of an allele pair based on comparison of their signals. The serotype comparison vector *Vs*, is based on grouped serotypes according to their average sequence similarities. Some serotypes are not very similar; however, they frequently remain together on the candidate list. We also consider those serotypes based on the observation of array analysis. Our data set contains 21 HLA-A serotypes distributed within nine serotype groups (serogroups), 36 HLA-B serotypes within 17 groups, and 14 HLA-C serotypes within seven groups. All possible serotypes that remain after the serotyping step are clustered according to the predefined serogroups. If a serogroup has two or more serotypes, we then generate a serotype comparison vector **Vs** for all the involved alleles; if there is only one serotype in a serogroup, we then generate an allele comparison vector **Va** for every allele in that serotype. Comparison vectors **Vs** and **Va** consist of all AvgWins(*x*) of every involved allele *x* and their number of negative probe signals. A serotype/allele comparison vector can be seen as a profile of the present serotypes and it represents the signature of the actual probe signals.

##### Knowledgebase search

The comparison vectors based on same alleles often share high correlation coefficients. Therefore, we can identify the actual alleles by using knowledgebase search and knowledge-based approach. The remaining serotypes are clustered according to the serotype groups. We generate a comparison vector for each serotype cluster. All the identified templates are ranked by their correlation coefficients, and only the top *N* (*N* ≤ 5) are used for genotyping of identified serotypes. If an allele is present in one of the templates, it is considered a possible allele. In most cases, the templates are very consistent with the alleles they represent. Only the present alleles in the templates are kept while others are removed. If a query serotype is not present, its profile can lead to templates with no present alleles. We demonstrated that the reproducibility is also observed on absent alleles. Based on the high reproducibility of array data, the accumulation of more samples and expanded representation of HLA alleles and genotypes in our knowledgebase, qualified templates for genotyping unknown samples has high probability of being correctly identified.

##### Knowledge-based approach

Given a serotype, if we cannot find any matching templates by doing a knowledgebase search, the knowledge-based approach is used to select possible alleles *x* based on the allele comparison result, AvgWins(*x*) and the number of negative probe signals, Neg(*x*). We define Weight(*x*) = AvgWins(*x*) − Neg(*x*) to represent the overall signal strength of *x* over the other alleles within the same serotype. If *x* is a present allele, we can expect a high value of Weight(*x*) due to relatively high AvgWins(*x*) and low Neg(*x*). On the contrary, if x is an absent allele, we can expect a low value of Weight(*x*). Each allele *x* is associated with a predefined WeightThreshold(*x*). If Weight(*x*) > WeightThreshold(*x*), then we assume x is a possible present allele. Based on a comprehensive data analysis on all the samples, we set an appropriate WeightThreshold(*x*) for each allele x.

### Ambiguity removal

There should be at most six alleles remaining after the analysis within Serotype Determination and Genotype Determination steps (two alleles for each HLA-A, -B, and -C, for a heterozygous sample). However, in some cases there is a remaining ambiguity resulting more than six HLA allele candidates. Such an ambiguity can result in multiple combinations of possible genotypes. In the Ambiguity Removal step we employ genotype comparison to select the best combination. A genotype of HLA Class I consists of at most two HLA-A, two -B, and two -C alleles. Based on this criterion, we generate all possible combinations of genotypes according to the remaining alleles, and perform a pairwise comparison for every pair of genotypes using the genotype comparison algorithm.

#### Genotype comparison algorithm

Genotype comparison algorithm is used to estimate the number of wins of a genotype pair based on comparison of their signals. Given two genotypes, *G*1 and *G*2, Sig(*G*1, *p*) represents the signal summation of all covered probes at position *p* of all involved alleles in *G*1, and Sig(*G*2, p) represent the signal summation of all covered probes at position *p* of all involved alleles in *G*2, and Max(*G*1, *G*2, *p*) is the larger of Sig(*G*1, *p*) and Sig(*G*2, *p*). We calculate the Wins(Gi) by comparing Gi with all the other genotypes, and assign Wins(Gmax) as the maximal score, then report all genotype combinations whose score above Wins(Gmax) − 20. All the methods and steps within the Computational Analysis have been automated and integrated into an HLA Class I genotyping automation.

## Results

### Probe and microarray design

We designed a microarray for highly parallel SSOP-based ultra-sensitive HLA typing. The probe set was derived from observed HLA sequences identified from the National Marrow Donor Program (NMDP) (17). In contrast to earlier microarrays that used a small number of ultra-specific probes designed to distinguish between HLA variants (20), we utilized the complete overlapping set of probes for target HLA sequences. The individual probes were shifted by a single nucleotide along the full length of HLA sequences. Each HLA array comprised 75,476 unique probes representing 1,610 full-length HLA class I A, B and C sequences (505 HLA-A, 703 HLA-B, and 402 HLA-C alleles, Table S2 in Supplementary material), and one negative control sequence. The design also included probes for 1,439 class II sequences and 77 HLA-E, F, and G alleles that were not analyzed in this study. A total of 175,611 probes were printed on each array. Initially, a starting overlapping set of 25 nucleotide-long probes were selected – each position in the 1,610 HLA class I sequences was represented by a unique probe. A representative probe is shown in Figure 2A (top). The melting temperature was calculated as follows:

$$\mathrm{Tm}=64.9+41\times \frac{y+z-16.4}{w+x+y+z}$$

Here, *w*, *x*, *y*, and *z* are the respective numbers of the bases A, T, G, and C in the probe, respectively (21).

The range of TMs of these probes had 23 discrete values between 41.3 and 77.4°C (Figure 2A, middle). Probe lengths were adjusted to target melting temperature of Tm = 64.2°C. Each probe was either extended or shortened to make its Tm as close as possible to the target temperature (Figure 2A, bottom). The adjusted length was 20–60 nucleotides, mandated by the Agilent SurePrint technology (Agilent, Santa Clara, CA, USA) (22). We used 60-mer SurePrint G3 Human CGH Microarray Kit, 4 × 180 K format to custom-design the microarrays. Probes were synthesized directly from dNTPs on the array surface. Each slide had four arrays (Figure 2B, left image). The array was designed using Agilent eArray platform and manufactured. Microarrays were printed on a standard (25 mm × 75 mm) glass slides.

### HLA determination

Of 21 blood samples, 19 were correctly typed for all loci (Table 3). Only two of 126 HLA alleles were incorrectly assigned in the blood samples. The two incorrect assignments (sample ID: 4 and 13) included the assignment of A*02:01/A*02:16 to an A*02:01 homozygous sample, and the assignment of C*07:01 to a C*07:06 sample. The alleles A*02:01 and A*02:16 have only two different nucleotides at positions 559-60 (AC/GA). The alleles C*07:01 and C*07:06 only have one different nucleotide at position 1,061 (C/T). Five samples (2, 6, 9, 11, and 13) were flagged as homozygous and three samples were flagged to contain closely related alleles (4, 6, and 7) meaning that these should undergo validation sequencing.

HLA typing of 18 cell lines (Table 4) included eight from the International Histocompatibility Working Group (IHWG) anthropology panel. This panel represented a broad diversity of world samples and contained several rare alleles (frequency < 0.1%, e.g., C*07:06, B*39:09, B*27:09, B*44:04, C*07:18, C*12:05, and C*15:04, respective samples 26, 33, 37, 34, 30, and 25) and several samples that contained closely related alleles (samples 22, 28, and 35 with B44, C7, and B27, respectively). Seven samples (13, 24, 25, 27, 29, 31, 35, and 36) were homozygous for at least one locus. Excluding A*24:33 (probes not present on the array), three of the 107 alleles were incorrectly typed, all of them flagged for validation sequencing.

**Table 4:** **Cell line samples used in this study**.

Sample	DFCI-ID	HLA-A	HLA-A	HLA-B	HLA-B	HLA-C	HLA-C	Sample name	Status/actual
22	40	26:01	29:02	Nil	44:03	05:01	16:01	1362-8572 (I)	B*44:02/44:03
23	29	01:01	24:02	08:01	15:01	03:03	07:01	FH15 (I)	OK
24	31	02:04	Nil	51:01	Nil	15:02	Nil	RML (I)	OK
25	35	24:02	Nil	15:17	51:01	07:01	15:04	BRIP (I)	OK
26	37	24:02	29:02	39:09	44:03	07:02	16:01	FH24 (I)	OK
27	38	29:02	Nil	14:02	57:01	06:02	08:02	MYE 2004 (I)	OK
28	39	01:01	02:01	07:02	08:01	07:01	Nil	FH53 (I)	C*07:01/07:02
29	43	Nil	11:01	37:01	58:01	03:02	06:02	WUZH1 (I)	OK
30	44	11:01	Nil	27:06	51:06	03:04	12:05	FH46 (I)	HLA-A*24:33
31	23	02:16	03:01	Nil	51:01	07:04	15:02	TUBO (A)	OK
32	26	31:01	33:01	14:02	35:02	04:01	08:02	FH3 (A)	OK
33	27	02:01	29:02	27:09	44:03	01:02	16:01	FH5 (A)	OK
34	30	02:05	68:02	14:02	58:01	07:18	08:02	FH1 (A)	OK
35	32	01:01	Nil	Nil	27:05	02:02	Nil	FH4 (A)	B*27:03/27:05
36	33	02:01	Nil	35:03	Nil	12:03	Nil	KOSE (A)	OK
37	42	01:01	68:01	15:01	44:04	06:02	16:01	1347-4843 (A)	OK
38	46	02:03	11:01	38:02	46:01	07:02	12:02	LCK (A)	OK
39	21	02:01	31:01	40:01	51:01	01:02	03:04	T1	OK

*Cell line sample are from the IHWG anthropology panel, designated by (A), or other IHWG cell lines (I). The correct HLA typing is in black font, the incorrect typing is shown in red. The actual alleles, if incorrectly assigned, are shown in column Status/Actual, or designated OK if correct*.

In addition to two frozen blood samples that were repeated in four arrays (Table 3), nine cell lines were typed as repeats, ranging from 1 to 3 repeats, a total of 24 arrays (Table 5). The first instance of triple homozygous cell line AMALA (sample 48, C*03:03) was mistyped as HLA*C*03:03/12:02, while subsequent repeats were correctly typed. All typing errors were in homozygous cell lines or heterozygous cell lines that have closely related HLA alleles that differ by up to three nucleotides. Typing accuracy of heterozygous samples was 100%. The typing error sources were:

- Heterozygous samples with two highly similar variants – samples 22, 28, and 35 were mistyped as homozygous.
- Homozygous samples with probe masking – e.g., sample 4 homozygous at A locus for A*02:01 had signals from B and C allele probes identical to HLA-A*02:16, resulting in A*02:01/02:16 assignment (Figure 2D).
- Mistaken assignment within the same serotype, e.g., sample 13 that has C*07:06 was assigned *07:01.
- Wrong allele of other serotype assigned to homozygous samples, such as sample 48 where C*03:03/12:02 was assigned to a homozygous C*03:03 sample.
- Missing allele because the probes do not exist on the array – sample 30 was incorrectly typed because probes specific for HLA-A*24:33 were not included in the array.
- Heavily degraded samples will result in typing errors. Sample 68 (Table S2 in Supplementary material) that has C*06:02/18:01 was assigned C*04:01/06:02. This sample had a very low mean signal, three times lower than an average sample (829 relative fluorescence units “RFU” vs. observed average of 2,519 RFU).
- Mixed samples will almost always result in typing errors, except when two homozygous samples are mixed – Table S2 in Supplementary material.

**Table 5:** **Cell line repeats used in this study**.

Sample	DFCI-ID	HLA-A	HLA-A	HLA-B	HLA-B	HLA-C	HLA-C	Sample name	Status/actual
40	22	02:01	25:01	13:02	38:01	06:02	12:03	LAZ509	OK
41	53	02:01	25:01	13:02	38:01	06:02	12:03	LAZ509 rep 1	OK
42	54	02:01	25:01	13:02	38:01	06:02	12:03	LAZ509 rep 2	OK
43	57	02:01	25:01	13:02	38:01	06:02	12:03	LAZ509 rep 3	OK
44	50	30:01	Nil	13:02	Nil	06:02	Nil	LBF	OK
45	65	30:01	Nil	13:02	Nil	06:02	Nil	LBF rep 1	OK
46	74	30:01	Nil	13:02	Nil	06:02	Nil	LBF rep 2	OK
47	77	30:01	Nil	13:02	Nil	06:02	Nil	LBF rep 3	OK
48	24	02:17	Nil	15:01	Nil	03:03	12:02	AMALA	C*03:03/03:03
49	63	02:17	Nil	15:01	Nil	03:03	Nil	AMALA rep 1	OK
50	69	02:17	Nil	15:01	Nil	03:03	Nil	AMALA rep 2	OK
51	49	03:01	11:01	40:01	Nil	03:04	Nil	FH14 (A)	OK
52	34	03:01	11:01	40:01	Nil	03:04	Nil	FH14 rep	OK
53	45	02:02	02:05	15:01	49:01	03:03	07:01	1416-1188 (A)	OK
54	60	02:01	02:05	15:01	49:01	03:03	07:01	1416-1188 rep	OK
55	28	30:01	68:02	Nil	42:01	17:01	Nil	RSH (A)	OK
56	70	30:01	68:02	42:01	Nil	17:01	Nil	RSH rep	OK
57	36	66:01	Nil	38:01	Nil	12:03	Nil	TEM	OK
58	71	66:01	Nil	38:01	Nil	12:03	Nil	TEM rep	OK
59	41	02:01	11:01	27:02	35:03	02:02	12:03	LUCE	OK
60	47	02:01	11:01	27:02	35:03	02:02	12:03	LUCE rep 1	OK
61	76	02:01	11:01	27:02	35:03	02:02	12:03	LUCE rep 2	OK
62	61	02:06	02:07	46:01	Nil	01:02	08:01	T7526	OK
63	73	02:06	02:07	46:01	Nil	01:02	08:01	T7526 rep	OK

*Repeats are designated by “rep” in column Sample name. The correct HLA typing is in black font, the incorrect typing is shown in red. The actual alleles, if incorrectly assigned, are shown in column Status/Actual, or designated OK if correct*.

In summary, samples typed by our method to have two variants within the same serotype, or typed as homozygous should be validated by confirmatory typing using RNA or DNA sequencing. This will initially account for approximately 30% of the samples. The need for confirmatory sequencing will diminish as the number of templates in the knowledgebase increases.

### Reproducibility of results

The reproducibility was assessed using repeat samples (Table 5) and repeat probes (data not shown). The array-wide probe signals showed high reproducibility with the majority of repeats showing *r* > 0.975 for each pair of arrays hybridized with the same samples (31 of 47) and *r* ≤ 0.975 for the arrays with different samples (all 879 pairs) (Figure 3). Three negative control examples were correctly assigned (Table S2 in Supplementary material) while the A*02:01 transgenic mouse sample was identified as negative because array-wide signals were low. Only a single sample (sample 48) was mistyped in one of the three repeats (Table 5). This sample was homozygous for C*03:03 but was assigned C*03:03/C*12:02. The results indicate that the microarray technology and sample processing can be standardized and used in a high-throughput fashion. Array matching was done by calculating overall correlation coefficients between the query array signals and the template array signals stored in the knowledgebase. All array pairs having *r* > 0.975 represented identical samples; they were the highest matches within the set of templates. Rare examples where the array signals correlation coefficients were high, but were mismatches, were rejected by the serotype determination algorithm (Figure 3). These results indicate that as the knowledgebase of templates grows, the majority of the HLA typing will be directly readable from the template matching.

## Discussion

The high-throughput methods such as next generation DNA (12) and RNA (13) sequencing are alternative HLA typing approaches. DNA sequencing has advantages: sample preparation is simple, actual sequence can be directly read, and null alleles and previously unknown sequences can be identified. The disadvantages include long reads, long turnaround time, and multiplexing – samples are labeled and then mixed before they are read, creating potential errors. The cost of DNA sequencing is relatively high without multiplexing. RNA sequencing has advantages: actual coding sequences are read directly, turnaround time is shorter, cost is lower than DNA sequencing and new sequences can be identified. The disadvantages include multiplexing and some sequencing ambiguities. The advantages of microarray-based typing include rapid turnaround, individual handling of each sample, and the establishment of a knowledgebase that improves the quality of HLA typing as it grows. This method is amenable to automation. The disadvantages of microarray approach include the lack of ability to identify novel sequences and the need to deal with masking problems. Samples that are homozygous or have two closely related alleles need to be confirmed by sequencing.

Bone marrow transplantation (BMT) is a treatment of choice in a spectrum of hematological malignancies, aplastic anemia, immunodeficiencies, hemoglobulinopathies, and inherited diseases, such as metabolic disorders and osteopetrosis. Recent reports suggest that viral clearance from HIV-positive individuals can be achieved using the BMT (23). The success rate of allogeneic BMT has steadily increased over the last 40 years, largely due to the HLA matching between donor and recipient (3, 24–26).

Drug toxicity associations with HLA have been known for several decades (7). Apart from the B*57:01-associated Abacavir hypersensitivity syndrome (ASH) (27) there is a myriad of reported drug toxicity associations. The anticonvulsant Carbamazepine can cause Stevens–Johnson Syndrome in HLA-B*15:02, B*15:11, B*15:18, A*30:10, A*31:01, and C*07:04 in a population-specific manner (28–36). A detailed listing of HLA associations with drug toxicity is shown in Table S7 in Supplementary material (7, 37–47). Precision HLA typing is important because single amino acid differences often define functional haplotypes whose differences may result in serious consequences. For example B*27:05 confers susceptibility to spondyloarthropaties (48) while B*27:03 is protective. These two alleles differ at a single nucleotide position 247 (exon 2, see Figure 1B) that codes for tyrosine in B*27:05 and histidine in B*27:03 (Y83H). This change modifies pocket F that binds a major anchor of HLA ligands and result in different peptide repertoires for B*27:05 and B*27:03. Abacavir toxicity is observed in HLA-B*57:01 individuals but not in individuals with the related allele B*57:03 that differs in two nucleotide positions at positions 340 (exon 2) and 347 (exon 3) causing differential drug binding and a resultant change in peptide repertoire upon Abacavir binding to HLA-B*57:01 (49, 50). Similarly, a major conserved T-cell epitope from human papilloma virus E7 antigen is presented by A*02:01, but not by A*02:07 (common in Asians). These two alleles differ in nucleotide position 296 (exon 2) resulting in a single amino acid change (Y99C) affecting the binding of peptide primary anchor P2. These examples suggest that the mechanism underlying these associations can be traced to fine differences in the binding groove altering the repertoires of HLA-presented peptides. They illustrate the importance of the precision and accuracy of HLA typing since small differences, even a single amino acid substitution, can have profound functional and clinical effects.

Human leukocyte antigens associations have been studied in more than 100 diseases including autoimmunity, allergy, infections, and cancer. Strong associations have been shown for rheumatoid arthritis, type 1 diabetes, celiac disease, inflammatory bowel disease, multiple sclerosis, autoimmune thyroid disease, psoriasis, ankylosing spondylitis, systemic lupus erythematosus, juvenile reactive arthritis and vitiligo (28, 31, 32, 34, 35). HLA associations have been reported in response to vaccines and specific mechanisms have yet to be described. Lower level of measles antibodies were observed in the HLA-B7 supertype individuals (51). Frequencies of HLA-A11 and -A24 were higher in Hepatitis B vaccine non-responders than in the responder group (52). HLA is used as a marker in population studies (53), elucidation of ancestry (54), tracking human migrations (19), prenatal testing (55), forensic science (56), human evolution genetics studies (57), and host-pathogen co-evolution (2, 58).

Human leukocyte antigens is such an important biomarker that the entire human population should be tissue typed, similarly as blood types are determined today. Combining HLA data with clinical records will have profound effects for the control of a variety of diseases. HLA typing is particularly important in clinical studies that involve immunology, HLA-related diseases, various cancers, and infectious disease. Many drugs that have failed clinical trials because of side effects can be re-examined for possible HLA-related toxicity. We estimate that in 70% of samples direct matching will be achieved by comparison with the knowledgebase templates. The remaining 30% of samples will be further validated by targeted sequencing, typically using a single locus sequencing run. Clinical records with the individuals’ HLA will allow identification of HLA disease associations, drug toxicity, and other clinically relevant associations. HLA typing is another frontier that will be conquered by advanced biotechnologies with computational methods. Population-wide HLA typing will have significant implications for personalized care and the improvement of public health.

## Conflict of Interest Statement

The authors declare that the research was conducted in the absence of any commercial or financial relationships that could be construed as a potential conflict of interest.

## Supplementary Material

The Supplementary material for this article can be found online at http://www.frontiersin.org/journal/10.3389/fimmu.2014.00597/abstract
