# Supplementary material for: Human Leukocyte Antigen Typing Using a Knowledge Base Coupled with a High-Throughput Oligonucleotide Probe Array Analysis
Source: Front Immunol. 2014 Nov 27;5:597. doi: 10.3389/fimmu.2014.00597 (PMC4245923; doi:10.3389/fimmu.2014.00597)
Supplement: Supplementary file 1 [file Data_Sheet_1.PDF]

Supplementary Material

Human Leukocyte Antigen (HLA) typing using a knowledge base coupled with a high throughput oligonucleotide probe array analysis

Guang Lan Zhang[1,2], Derin B. Keskin[1,3,4], Hsin-Nan Lin[1,5], Hong Huang Lin[1,6], David S. DeLuca[1,7], Scott Leppanen[8], Edgar L. Milford[3,9], Ellis L. Reinherz[1,3,4], Vladimir Brusic[1,2,3 *]

[1] Cancer Vaccine Center, Dana-Farber Cancer Institute, Harvard Medical School, Boston, MA, USA
[2] Department of Computer Science, Metropolitan College, Boston University, Boston, MA, USA
[3] Department of Medicine, Harvard Medical School, Boston, MA, USA
[4] Laboratory of Immunobiology, Department of Medical Oncology, Dana-Farber Cancer Institute, Boston, MA, USA
[5] Institute of Information Science, Academia Sinica, Taipei, Taiwan
[6] Department of Medicine, Boston University School of Medicine, Boston, MA, USA
[7] Current address: Cancer Program, Broad Institute, Cambridge, MA, USA
[8] Agilent Technologies, Inc., Santa Clara, CA, USA
[9] Histocompatibility and Tissue Typing Laboratory, Brigham and Women's Hospital, Boston, MA, USA.

*Correspondence:
Vladimir Brusic, Cancer Vaccine Center,
Dana-Farber Cancer Institute, Harvard Medical School,
77 Avenue Louis Pasteur, HIM 401, Boston, MA 02115, USA
e-mail: vladimir_brusic@dfci.harvard.edu

1. Supplementary Figures and Tables

1.1. Suplementary Tables

Supplementary Table 1. Description of HLA alleles that were arrayed on the HLA chip. The set includes 505 genetic HLA-A variants (159 proteins), 703 -B (281 proteins), and 402 -C (123 proteins). The sequences of these alleles were extracted from the HLA-IMGT database (17).

Locus	Serotype	Alleles (number of variants if greater than 1)
A	*01:	01(42),02,03
A	*02:	01(61),02,03(4),04,05(5),06(10),07,08,09,10,11(4),12,13,14,16,17(2),19,20(2),21,22(2), 24(2),25,26,27,30,34,36,37,38,42,44,45,49,51,54,55,58,60(2),67,74(2)
A	*03:	01(32),02(2),05,07,08,10
A	*11:	01(32),02(3),03,04,05,06,10,12,13,19,20
A	*23:	01(7),02,05,09
A	*24:	02(51),03(2),04,05,06,07,08,10,13(2),14,17,18,20,21(2),22,23,25,26,28,29,31,35,46,51,58
A	*25:	01(5),02
A	*26:	01(22),02,03(2),05,07(2),08,09,10,12,16,18
A	*30:	01(5),02(5),03,04(2),06,08,09,10,11(2),12
A	*31:	01(9),02,03,04,06,08,09,12

**Supplementary Table S1 continued**		
A	*32:	01(11),02,03,05,06
A	*33:	01(6),03(7),05
A	*34:	01(2),02(2),03,05
A	*36:	01,03
A	*43:	01
A	*66:	01,02,03
A	*68:	01(11),02(6),03(3),04,05,07,13,15,16,17,23,25
A	*69:	01
A	*74:	01,03,04,09,11
A	*80:	01
**Locus**	**Serotype**	**Alleles (number of variants if greater than 1)**
B	*07:	02(28),04,05(6),06,07,09,10,13,14,15,20,21,22(2),33,36
B	*08:	01(16),02,03,04,05,12(3)
B	*13:	01(6),02(12),03,04,09,11,13
B	*14:	01(2),02(6),03,05,06(2)
B	*15:	01(23),02(5),03(3),04,05(2),06,07(2),08,09,10(2),11(5),12,13(2),14,15,16(3),17(3),18(4),20, 21,23,24,25(3),27(3),29,30,31,32,34,35,37,38(2),39(2),40,45,46,47,48,50,52,53,54,56,58, 61,63,70,71,86
B	*18:	01(14),02,03,04,06,07(2),08
B	*27:	01,02(2),03,04(3),05(16),06,07,08,09,10,11,12,13,14,20,21
B	*35:	01(30),02(4),03(10),04(3),05(2),06,08(4),09(2),10,11(2),12(3),13,14(2),15,16,17,18,19,20(2), 21,22,23,27,28,30,32,33,43(2),46
B	*37	01
B	*38:	01(5),02(3),04,05,06,09
B	*39:	01(11),02(2),03,04,05(2),06(2),07,08,09,10(2),11,12,13(2),14,15,24(2)
B	*40:	01(23),02(12),03,04,05,06(5),07,08,09,10(2),11(2),12,14(3),16,18,20,21,23,27(2),31,35,36, 37,38,39,40,42,44,49,50,52
B	*41:	01,02(4),03(2)
B	*42:	01(2),02
B	*44:	02(21),03(14),04,05(3),06,07,09,10,12,15,18,22
B	*45:	01,06
B	*46:	01(7),02
B	*47:	01(3),02,03
B	*48:	01(3),02(2),03(2),04,06,07,08
B	*49:	01(3),02
B	*50:	01(3),02
B	*51:	01(28),02(5),04,05,06(2),07(2),08,09(2),10,14,19,21,22,31,32,34,37
B	*52:	01(10)
B	*53:	01(5),02,04,08(2)
B	*54:	01(2),02
B	*55:	01(7),02(6),04,07,08,10,12,16
B	*56:	01(4),02,03,04,06,07,09,11
B	*57:	01(11),02(2),03(2),04,06
B	*58:	01(9),02,06
B	*59:	01(2)
B	*67:	01(2),02
B	*73:	01
B	*78:	01(2),02(2)
B	*81:	01
B	*82:	01,02

**Supplementary Table S1 continued**		
**Locus**	**Serotype**	**Alleles (number of variants if greater than 1)**
C	*83:	01
C	*01	02(14),03,04,05,06,07,08,10,16,18,30
C	*02	02(19),03,14,18
C	*03	02(9),03(16),04(24),05,06,07,08,09,12,13,15,16,17,19,36,37,38(2)
C	*04	01(36),03,04(2),05,06,07,08,10,13,14,15(3),27
C	*05	01(18),05,09
C	*06	02(17),03,04,06,08,09,10,15,24
C	*07	01(24),02(31),03,04(6),05,06,07,08,09,10,12,13,14,17,18,26,27(2),29,35,43,56(2),66,67
C	*08	01(5),02(6),03(2),04,05,06,11,12,13,20,21,27
C	*12	02(6),03(18),04(2),05,07,12
C	*14	02(7),03,04,06
C	*15	02(7),03,04,05(6),06(3),07,08,09,11,13,17,19,20
C	*16	01(6),02(8),04,08,
C	*17	01(7),03,04

Supplementary Table 2. Control samples used in this study include negative controls including A2 transgenic mouse (samples 64-67), a degraded sample derived from Merkel cell carcinoma (sample 68), and mixed samples (samples 69-72). The correct HLA typing is in black font, the incorrect typing is shown in red.

Sample	DFCI ID	HLA-A	HLA-A	HLA-B	HLA-B	HLA-C	HLA-C	Sample name	Status/Actual
**64**	**01**	nil	nil	nil	nil	nil	nil	Blank array	nil
**65**	**03**	nil	nil	nil	nil	nil	nil	Blank array	nil
**66**	**51**	nil	nil	nil	nil	nil	nil	A2mus	A*02:01
**67**	**52**	nil	nil	nil	nil	nil	nil	B6mus	nil
**68**	**48**	02:05	68:02	35:02	nil	18:01	06:02	Merkel	B*35:02/35:01<br>C*04:01/06:02
**69**	**55**	69:01	66:01	27:02	38:01	12:03	15:02	MIX LUCE + TEM	A*02:01/11:01/66:01<br>B*27:02/35:03/38:01<br>C*02:02/12:03
**70**	**56**	02:17	66:01	15:01	38:01	03:03	12:03	MIX AMALA + TEM	A*02:17/66:01<br>B*15:01/38:01<br>C*03:03,12:03
**71**	**58**	30:08	68:02	15:01	42:01	07:01	17:01	MIX RSH + 1416-1188	A*02:01/02:05<br>A*30:01/68:02<br>B*15:01/42:01/49:01<br>C*03:03,07:01,17:01
**72**	**64**	02:17	nil	46:01	nil	03:03	08:01	MIX AMALA + T7526	A*02:06/02:07/02:17<br>B*15:01/46:01<br>C*01:02/03:03/08:01

Supplementary Table 3. The ranges of measured raw signals across arrays: Min – minimum signal, Max – maximum signal, Mean – the mean value of the signal. Minimum raw signals ranged from 2 to 11, Maximum signal ranged from 64,948 to 770,759, and mean signal ranged from 239 to 4,181. Normalization of signals puts all microarrays on a common scale 1-20,000 with the array-wide mean signal of 1,000.

Array	Min	Max	Mean	Array	Min	Max	Mean	Array	Min	Max	Mean
**001_1**	3	284,494	829	**007_1**	5	275,882	3,484	**017_1**	4	200,046	2,846
**001_2**	3	238,340	2,899	**007_2**	5	264,679	3,028	**017_2**	3	156,945	1,921
**001_3**	3	438,534	2,244	**007_3**	4	428,657	2,889	**017_3**	2	64,948	429
**001_4**	4	467,674	2,815	**007_4**	4	333,153	3,857	**017_4**	3	251,059	4,716
**002_1**	9	275,555	3,690	**008_1**	6	287,272	2,427	**018_1**	3	256,847	2,708
**002_2**	3	277,316	3,003	**008_2**	7	698,462	3,421	**018_2**	2	230,038	2,022
**002_3**	3	243,403	2,808	**008_3**	11	309,718	4,181	**018_3**	2	236,461	1,960
**002_4**	3	242,756	3,464	**008_4**	9	308,233	3,490	**018_4**	2	241,541	3,208
**003_1**	4	122,049	336	**013_1**	4	294,820	2,842	**019_1**	3	322,914	4,704
**003_2**	4	231,988	2,905	**013_2**	4	272,212	9	**019_2**	2	240,875	2,291
**003_3**	3	113,693	571	**013_3**	3	269,749	3,486	**019_3**	2	247,464	2,155
**003_4**	3	421,677	3,138	**013_4**	3	403,678	9	**019_4**	3	239,824	1,884
**004_1**	6	442,678	3,697	**014_1**	6	78,430	2,725	**020_1**	4	552,216	2,956
**004_2**	3	249,733	1,572	**014_2**	3	77,794	2,866	**020_2**	3	246,279	2,905
**004_3**	4	263,031	2,777	**014_3**	3	76,326	2,544	**020_3**	2	342,309	2,365
**004_4**	4	275,238	3,092	**014_4**	3	82,739	3,546	**020_4**	2	770,759	2,962
**005_1**	4	270,992	3,458	**015_1**	2	103,875	801	**021_1**	3	221,990	1,722
**005_2**	3	260,797	2,475	**015_2**	2	87,802	486	**021_2**	3	238,556	2,005
**005_3**	3	294,860	2,112	**015_3**	2	47,174	282	**021_3**	2	121,057	914
**005_4**	4	318,514	3,422	**015_4**	2	192,840	2,550	**021_4**	2	214,403	1,231
**006_1**	9	529,450	2,885	**016_1**	2	93,220	239	**022_1**	2	85,696	3,113
**006_2**	3	288,366	2,644	**016_2**	2	250,126	954	**022_2**	2	79,662	2,842
**006_3**	4	511,408	3,308	**016_3**	4	237,626	3,738	**022_3**	2	75,143	1,381
**006_4**	7	377,314	2,645	**016_4**	2	182,896	2,375	**022_4**	2	84,823	2,093

Supplementary Table 4. Ranks of the correct serotypes across all studied arrays.

Rank	Number of serotypes present in the samples
**1**	241
**2**	155
**3**	51
**4**	20
**5**	4
**6**	1

Supplementary Table 5. Maximal number of negative signals for HLA class I serotypes. Serogroups were identified by the analysis of similarity of allele sequences. The values of MaxNeg were determined empirically from sample serotyping.

Serotype	Serogroup	MaxNeg	Serotype	Serogroup	MaxNeg	Serotype	Serogroup	MaxNeg
A01	A1	40	B08	B8	30	B42	B42	20
A36	A1	30	B13	B13	30	B44	B44	40
A02	A2	40	B14	B14	20	B45	B45	30
A68	A2	40	B38	B14	30	B49	B45	20
A69	A2	30	B39	B14	30	B50	B45	20
A03	A3	30	B67	B14	NA	B57	B57	20
A11	A3	30	B15	B15	25	B58	B57	20
A23	A24	30	B46	B15	20	B73	B73	40
A24	A24	30	B18	B18	20	B83	B83	40
A25	A25	30	B27	B27	40	C01	C1	10
A26	A25	30	B35	B35	20	C02	C2	10
A34	A25	30	B51	B35	25	C03	C3	30
A43	A25	30	B52	B35	20	C04	C3	20
A66	A25	30	B53	B35	20	C14	C3	10
A29	A29	30	B54	B35	20	C15	C3	20
A30	A30	40	B55	B35	20	C16	C3	20
A31	A31	30	B56	B35	20	C18	C3	20
A32	A31	30	B59	B35	20	C05	C5	20
A33	A31	30	B78	B35	20	C08	C5	20
A74	A31	30	B37	B37	30	C06	C6	20
A80	A31	40	B40	B40	10	C12	C6	30
B07	B7	30	B47	B40	10	C07	C7	40
B48	B7	20	B41	B41	20	C17	C7	40
B81	B7, B35	20						

Supplementary Table 6. The values of MaxDifference for selected serotype comparisons. Because of the small number of differences, these combinations of serotypes cannot be distinguished by the MaxDifference measure and need to be further analyzed.

Serotype1	Serotype2	MaxDifference
A*01	A*36	10
A*02	A*69	5
B*15	B*46	0
C*03	C*14	0

Supplementary Table 7. HLA Class I associations with drug-induced toxicity. Drugs are grouped by adverse reaction types. G1: Stevens-Johnson syndrome (SJS) and toxic epidermal necrolysis (TEN). G2: Drug-induced hypersensitivity syndrome (DIHS), drug reaction with eosinophilia, and systemic symptoms. G3: Fixed drug erruptions. G4: DIHS/liver. G5: Agranulocytosis.

			HLA associations			
**Group**	**Drug**	**Type**	**HLA-A**	**HLA-B**	**HLA-C**	**References**
G1	Allopurinol	anti-hyperuricemic		58:01		7
G1	Carbamazepine	anticonvulsant	30:10, 31:01	15:02/:11/:18, 59:01	07:04	7, 35
G1	Oxcarbazepine	anti-epileptic		15:02/:18		36, 42
G1	Phenytoin	anti-epileptic		13:01, 15:02	08:01	7, 35-37
G1	Oxicam	anti-inflammatory	A02	B44/45, B73		7, 38-41
G1	Sulfamethoxazole	Antibiotic	A29	B38, B44/45		7
G1	Methazolamide	carbonic anhydrase inhibitor		59:01	01:02	41
G1	Sulphonamides	antibiotics, antidiuretics	A29	B44/45		38
G2	Abacavir	antiretroviral		57:01		7
G2	Aminopenicillin	antibiotic	A02			7
G2	Nevirapine	antiretroviral		B14, B64/65, 35:05	C04, C08	7
G2	Lamotrigine	anti-epileptic	68:01	B38, 58:01,		7
G2	Trichloroethylene	industrial solvent		13:01		7
G3	Co-trimoxazole	antibiotic	A30	B13	C06	7
G3	Febrazone	analgesic		B54/55/56		43
G4	Flucloxacillin	antibiotic		57:01		7
G4	Ticlopidine	anti-platelet	33:03			7
G4	Amoxicillin	antibiotic	02:01			34
G5	Dipirone	Analgesic	A24	B07		38
G5	Levamisole	anthelminthic		B*27		38
G5	Clozapine	Antipsychotic		B38		44

1.2. Suplementary Figures

POSITION	1	2	3	4	5	6	7	8	9	10	11	12	13	14	15	16	17	18	19	20
A* THRESHOLD	40	44	76	514	992	735	2000	2000	2000	2000	2000	2000	2000	2000	1549	904	433	226	174	177
A* THRESHOLD CORRECTED	40	164	288	412	536	660	782	1116	1441	1784	2118	2452	2786	4000	4000	4000	2905	1794	1518	1534
A*01:01:01:01 SIGNAL	408	385	444	481	569	695	932	2448	2381	3094	3440	7895	20000	20000	15493	8869	4330	2269	1748	1771
A*02:01:01:01 SIGNAL	408	385	444	481	569	695	632	541	501	870	860	1248	2124	2518	2243	1680	1480	1319	1288	1297

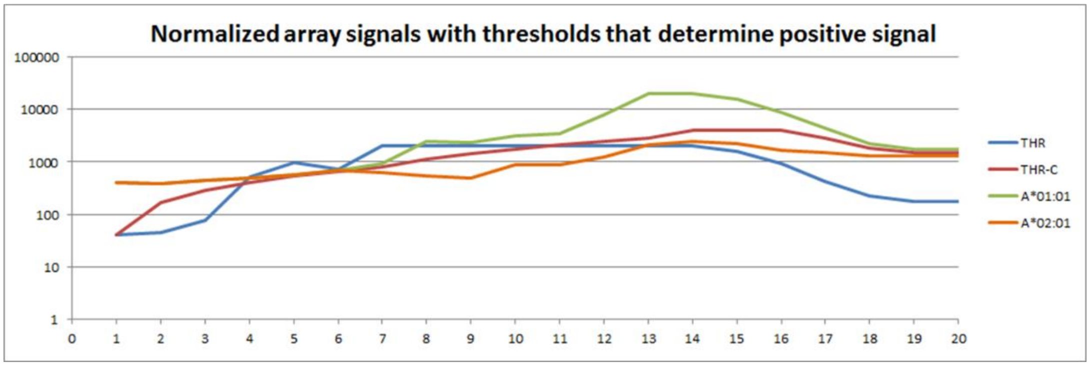

Supplementary Figure 1. Signals of overlapping probes. HLA-A*01:01 was present and HLA-A*02;01 was absent from this sample. The position 5 and 7 with signals of 569 and 932, respectively are the actual positive signals for HLA-A*01:01. The positions 8-20 represent positive signals for A*01:01 and negative signals for A*02:01. The theoretical threshold (10% of maximal signal for each position across all arrays) is shown as THR. The corrected threshold based on observed measurements is shown as THR-C.The figure shows normalized corrected data.

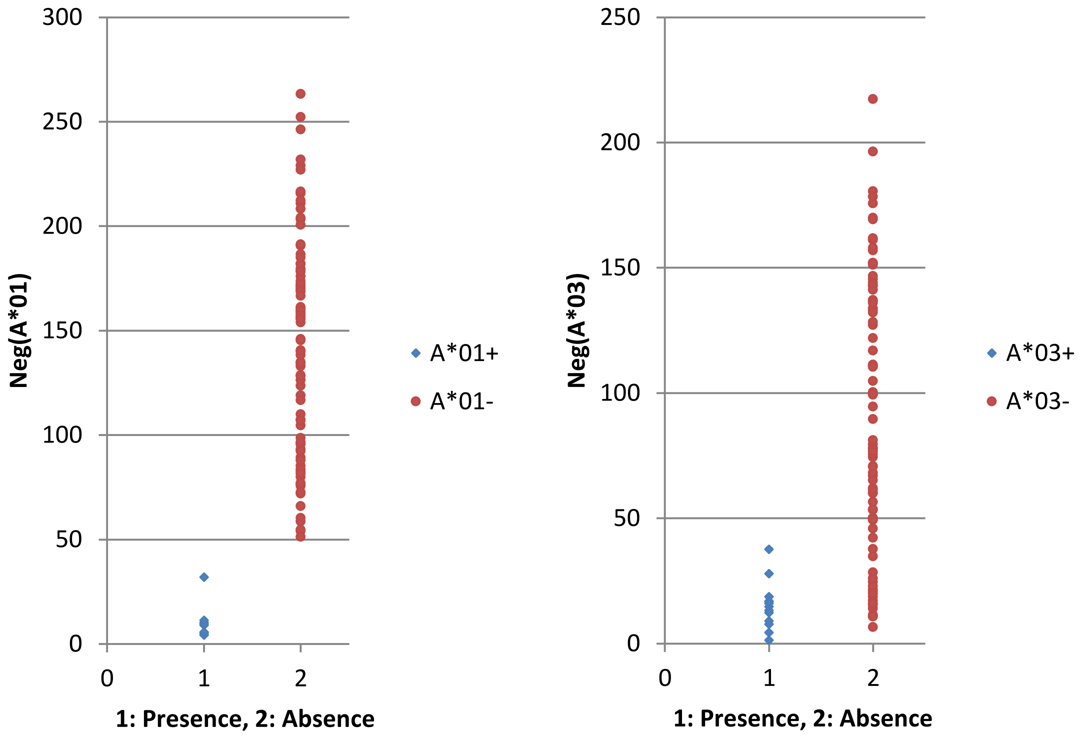

Supplementary Figure 2. Representative examples of discrimination between positive and negative samples for a particular serotype. The Neg(A*01) distribution of all samples is shown on the left. A*01+ represents Neg(A*01) with a present A*01, and A*01- represents Neg(A*01) with an absent A*01. There is a clear gap between Neg(A*01+) and Neg(A*01-). The Neg(A*03) distribution of all samples is shown on the right. A*03+ represents Neg(A*03) with a present A*03 and A*03- represents Neg(A*03) with an absent A*03. Some of Neg(A*03-) overlap with Neg(A*03+) due to probe masking between A*03 and A*11.
